# The dual role of Nrf2 in melanoma: a systematic review

**DOI:** 10.1186/s12860-023-00466-5

**Published:** 2023-02-06

**Authors:** Zahra Malakoutikhah, Zahra Mohajeri, Nasim Dana, Shaghayegh Haghjooy Javanmard

**Affiliations:** Applied Physiology Research Center, Cardiovascular Research Institute, Isfahan University of Medical Sciences, Isfahan, Iran

**Keywords:** Melanoma, Nrf2, Chemoresistance, Metastasis, Oxidative stress, Systematic review

## Abstract

Melanoma is the most lethal type of skin cancer that originates from the malignant transformation of melanocytes. Although novel treatments have improved patient survival in melanoma, the overall prognosis remains poor. To improve current therapies and patients outcome, it is necessary to identify the influential elements in the development and progression of melanoma.

Due to UV exposure and melanin synthesis, the melanocytic lineage seems to have a higher rate of ROS (reactive oxygen species) formation. Melanoma has been linked to an increased oxidative state, and all facets of melanoma pathophysiology rely on redox biology. Several redox-modulating pathways have arisen to resist oxidative stress. One of which, the Nrf2 (nuclear factor erythroid 2-related factor 2), has been recognized as a master regulator of cellular response to oxidative or electrophilic challenges. The activation of Nrf2 signaling causes a wide range of antioxidant and detoxification enzyme genes to be expressed. As a result, this transcription factor has lately received a lot of interest as a possible cancer treatment target.

On the other hand, Nrf2 has been found to have a variety of activities in addition to its antioxidant abilities, constant Nrf2 activation in malignant cells may accelerate metastasis and chemoresistance. Hence, based on the cell type and context, Nrf2 has different roles in either preventing or promoting cancer. In this study, we aimed to systematically review all the studies discussing the function of Nrf2 in melanoma and the factors determining its alteration.

## Background

Melanoma is a potentially fatal type of skin cancer caused by the uncontrolled proliferation of melanocytes [1]. Melanoma is responsible for more than 80% of skin cancer mortality. Its incidence has increased dramatically over the world in the last 50 years in all age groups, and based on incidence curves, the incidence will grow sharply in the coming years [2]. Although novel treatments such as targeted and immune therapies have improved patient survival in melanoma, chemoresistance prevents improving patients’ survival. The prognosis is still unsatisfactory, with most patients relapsing soon after their initial response [3]. As a result, new therapeutic targets must be identified to improve existing therapies and the prognosis of refractory melanomas.

Melanin synthesis comprises oxidation reactions and the generation of ROS (reactive oxygen species) by melanocytes, such as superoxide anions and hydrogen peroxide. These reactive oxygen species have a high tendency to react with DNA, lipids, and proteins, causing cell death. Even while oxidative species are synthesized, an advanced antioxidant system, paracrine agents, and gene regulatory networks keep them at a low level to prevent oxidative species-induced cellular damage. If antioxidants do not detoxify ROS, the cell will experience a rise in oxidative stress, altering the structure and function of critical cellular macromolecules like DNA, leading to excessive cell proliferation, mutation, and/or chromosome instability, and eventually the development of neoplasms. Oxidative stress and the consequent oxidative damage have emerged as key determinants involved in all stages of melanoma development. Redox signaling is disrupted by oxidative imbalance, and metabolic remodeling occurs in addition to mitochondrial and nucleus genetic instability. These events play an important role in tumor growth, metastasis, and the emergence of chemoresistance [4–9]. One of the most essential proteins involved in controlling antioxidant response is the transcription factor Nrf2 (nuclear factor erythroid 2-related factor 2), also known as the master regulator of antioxidant and cytoprotective genes. Nrf2 is located in the cytosol in physiological states, coupled to its inhibitor Keap1 (Kelch-like ECH-associated protein), resulting in deactivation via ubiquitination and proteasomal destruction. When Keap1 is subjected to oxidative stress, its cysteine residues oxidize, causing it to alter shape. Nrf2 is released, translocated into the nucleus, binding to ARE (antioxidant response element) sequences in the promoter of genes coding for antioxidant enzymes such as HO-1 (heme-oxygenase-1), SOD (superoxide dismutase), CAT (catalase), and GPX (glutathione peroxidase) [10, 11]. Thus, through the up-regulation of downstream genes, Nrf2 inhibits carcinogenesis by assuring rapid enzymatic change and neutralization of various carcinogens, as well as suppressing ROS or repairing oxidative damage [12, 13].

On the other hand, the endogenous antioxidant system adjusts with elevated oxidative stress following cancer initiation, leading to altered redox homeostasis. According to emerging data, the Nrf2 signaling pathway has been shown to enhance tumor cell proliferation, migration, invasion, angiogenesis, as well as radio/chemotherapy resistance.

So, depending on the cell type and circumstance, Nrf2 appears to be a double-edged sword [14]. The involvement of Nrf2 and its downstream target genes in melanoma will be discussed in this systematic review, indicating a dual role of Nrf2 as both oncogene/onco-suppressive protein.

## Materials & method

### Literature search strategy

The current systematic review followed PRISMA principles. Two researchers evaluated the search results independently to minimize the bias. Searches were conducted until May 2022, using three databases, including Web of Science (WOS), PubMed, and Scopus. On these databases, we searched the keywords ‘melanoma’ AND “Nuclear Respiratory Factor 2’ OR ‘Nuclear Factor Erythroid 2 Like 2’ OR ‘NFE2L2’ OR ‘Nrf2” and other related MeSHs in title, abstract, or keywords. We modified the search keywords to get the optimal results using the Scopus database, searching only ‘melanoma’ AND “NFE2L2’ OR ‘Nrf2”. All titles and abstracts were screened to find relative articles that met the inclusion criteria for full-text screening.

### Study selection

We used the EndNote reference manager to collect search results for initial assessment and exclusion of duplicate articles. Articles selected for review included the following inclusion criteria; (I) English language, (II) the title/abstract indicating the relationship between melanoma and Nrf2 or related genes. In addition, exclusion criteria are books, documents, and reviews. After excluding irrelevant articles, the remaining articles’ full text was retrieved for further assessment.

### Results analysis

Two researchers screened the search results independently. Study selection was performed based on the relevancy, inclusion, and exclusion criteria. The critical data extracted from each article included (I) first author name, (II) publication year, (III) location of study, (IV) study subject, (V) preliminary results, and (VI) conclusion (Table 1). Studies on the correlation of Nrf2 and melanoma cells were the primary outcome of interest. Regarding many heterogeneous study subjects and different study designs and protocols, conducting a meta-analysis was not deemed to be possible.

**Table 1:** The summary of the final selected studies

Study	Sample	Primary results	Conclusion	References
Choi et al., 2014	The human melanoma cell line G361	Although Nrf2 expresses in normal skin cells, it is downregulated in skin cancers, including malignant melanoma. However, the Keap-1 expression varies in normal cells and skin cancers, suggesting that Nrf2 translocation to nucleus may occur through Keap-1 independent pathways in human skin cancers.	The Nrf2/Keap1 pathway dysregulation relates to skin cancers, including melanoma.	[15]
Funes et al., 2014	Human mesenchymal stem cells, and human mammary epithelial cells	The intracellular level of ROS elevates during human cell transformation, which accompanied by Nrf2 repression through stimulation of RAS/RAF/ERK signaling pathway. Interestingly, regeneration of Nrf2 level in transformed cells elicits antioxidant responses and inhibit tumor growth.	Nrf2 expression is suppressed in some types of cancers. However, there are no significant differences between normal and melanoma cells in the NFE2L2 expression. Down-regulation of NFE2L2 is associated with poor prognosis in melanoma.	[16]
Smith et al., 2014		NOX activity has a crucial role in UVA-induced oxidative stress. UV radiation leads to Nrf2 protein accumulation as well as Nox1 protein expression and NOX activity. Overexpression of Nrf2 and Nox1 has been observed in all melanoma cell lines and tissue samples.	Redox imbalance following UVR favors the condition for melanoma formation. Co-activation of oxidant and antioxidant responses leads to tumor growth and progression.	[17]
Shinpei et al., 2014		Constitutive activation of Nrf2 has a significant role in emerging melanoma-resistant cells.	Intracellular ROS levels are significantly higher in Nrf2 knockout cells than in wild-type cells, sensitizing the melanoma cells to cisplatin or dacarbazine.	[18]
Hambright et al., 2015	Human melanoma cell lines: MeWo, WM793B, and 1205Lu, human epidermal neonatal melanocytes (HEMn)	Nrf2 protein level was higher in all melanoma cell lines than HEMn. NQO1 was meaningfully increased in WM793B cell lines, and *HMOX1* transcript was higher in MeWo and 1205Lu than HEMn. Basal oxidative stress in melanoma cells is higher than in normal melanocytes, consequently up-regulating the Keap1/Nrf2 signaling pathway to adapt to this oxidative stress. The elevated oxidative stress inhibits the dependency of melanoma cell survival on PI3K/AKT/mTOR pathway.	The oxidative stress level is directly related to an advanced stage of melanoma and metastasis. Redox therapeutic agents inhibit cell growth and survival by disrupting PI3K/AKT/mTOR pathway.	[19]
Hintsala et al., 2016	Human tissue sample; Cell lines: COLO-800, SK-MEL-1 with BRAF mutation, SK-MEL-30, and IPC-298 with NRAS mutation	In melanoma cells with BRAF\NRAS mutations, administration of their inhibitors would decrease the expression of Nrf2. Nuclear expression of Nrf2 in melanoma correlates with higher Clark level, deeper Breslow index, nodular growth, and poor survival.	Nrf2 is an influential factor in determining the prognosis of melanoma patients.	[20]
Chaiprasongsuk et al., 2016	Primary human epidermal melanocytes (HEMn), B16-F10 mouse melanoma cells	Knockdown of the Nrf2 gene in B16-F10 and HEMn cells results in melanogenesis enhancement in response to UVA exposure. UVA irradiation affects Nrf2 nuclear accumulation, and Nrf2 target antioxidants time-dependently.	Nrf2 has a protective role against melanogenesis under UVA irradiation.	[21]
Kasai et al., 2016	Cell lines; A7, C32, GAK, G-361, HMY-I, HMV-II, MM-AN, MeWo, SK-MEL-2, SK-MEL-28 and SK-MEL-31, nine non-small cell lung cancer cell lines, normal human dermal fibroblasts, and neonatal skin fibroblasts	A high proportion of NQO1-high melanomas present Nrf2 activation independent of KEA1 mutation. Melanoma cell lines presenting NQO1 overexpression show sensitivity to 17-AAG compared to cell lines with low expression of NQO1.	17-AAG is a potential therapeutic option for NQO1-high melanoma.	[22]
Saddawi-Konefka et al., 2016	Mice cell lines: MCA-induced sarcoma, Ramos, B16, and LLC	Tert-butylhydroquinone (tBHQ) induced activation of Nrf2 results in elevated expression of il17d transcript in the B16 cell line. IL-17D induction leads to NK cell-dependent tumor inhibition.	Activation of the Nrf2-IL17D pathway in primary tumorigenesis leads to NK cell-dependent tumor inhibition. However, the role of the Nrf2-IL17D axis is context-dependent and may not always associate with NK infiltration or better survival. Nrf2 stimulates both internal and external anti-tumor immune responses, prevents tumor progression, and improves survival.	[23]
Zhu et al., 2016	Nrf2-null C57BL/6 mice, and male Nrf2-null and wild-type mice Cell line: B16-F10-luc-G5	Nrf2 deficiency results in tumor growth and a higher risk of lung metastasis in B16-F10 melanoma cells.	The exact role of the Nrf2 pathway in carcinogenesis needs to be investigated. However, diverse factors such as type of carcinogens, cancer type, and host immunity appear to contribute to the oncosuppressor/oncogene role of Nrf2.	[24]
Rocha et al., 2016	Human melanoma cell lines: SKMEL 28 and SKMEL 94 Murine melanoma B16 human glioma cell lines: U138MG and U87MG	Temozolomide (TMZ) increases the intracellular ROS level and triggers Nrf2 and its downstream GCLM and GSTπ genes. Consequently, the elevated level of GSH mediates temozolomide resistance. In addition, GSH depletion increases melanoma cells’ sensitivity to temozolomide. Nrf2 silencing results in temozolomide-induced cell death in vitro and in vivo.	Nrf2 is a key factor in developing TMZ drug resistance in melanoma.	[25]
Benlloch et al., 2016	Female nu/nu nude mice, Human epidermal melanocytes: HEMa-LP, pancreatic adenocarcinoma: ASPC-1 and BxPC-3, Human A2058, MeWo, and MelJuso melanoma cells, murine B16 melanoma F1, mouse pituitary corticotroph tumor cells: AtT-20	Pterostilbene indirectly downregulates Nrf2 and the downstream antioxidant defense system by inhibiting ACTH production, and reducing plasma levels of corticosterone, thus restricting melanoma tumor growth. In contrast, genetically induced Nrf2 overexpression or administration of exogenous corticosterone hindered tumor growth suppression of melanoma cells.	Pterostilbene prevents human melanoma growth in vivo. However, it does not affect melanoma growth in vitro at levels tested within the tumors. The anti-tumor effect of pterostilbene is dependent on the Nrf2 signaling pathway.	[26]
Cai et al., 2017	Melanoma patient (case report)	A variation of NFE2L2 (NFE2L2 p.Leu266Phe) is identified in a patient with stage III metastatic melanoma.	NFE2L2 mutation may play a role in melanogenesis and cancer progression.	[27]
Hintsala et al., 2017	Human tissue sample	The nuclear expression of Nrf2 is significantly higher in metastatic melanoma than in primary melanoma, whereas the level of cytoplasmic Keap1 in metastatic melanoma is lower than in primary melanoma.	Nuclear expression of Nrf2 is associated with distant metastasis and poor prognosis.	[28]
Zhao et al., 2017	WT/Sesn2 KO mouse embryonic fibroblast, A375, iMC23 melanocytes, and normal human epidermal melanocytes	UV irradiation induces Sesn2 expression in melanoma cells. Sesn2 silencing stimulates UVA-induced Nrf2 activation, inhibiting UVA-induced ROS generation. Nrf2 silencing does not affect UVA-induced expression of Sesn2 in A375 cells.	Sesn2 seems to be the upstream suppressor of Nrf2 in response to UVA exposure.	[29]
Gao et al., 2018	B16-F10 melanoma cells	UV radiation induces Nrf2 and caspase-3 expression in B16-F10 melanoma cells, decreasing cell viability. However, Inhibition of Nrf2 by siRNA also leads to a decrease in cell viability, invasion, and migration of irradiated melanoma cells and an increase in caspase 3 expression.	Nrf2 signaling pathway exerts a dual role in melanoma. The effect of UV on the Nrf2 signaling pathway is dose-dependent.	[30]
Khamari et al., 2018	Tumor specimens, A375 melanoma cell line	A375 melanoma cells present Nrf2 nuclear accumulation, notably higher in BRAFi-resistant cells. Knockout of Nrf2 in BRAF-resistant melanoma cells reversed vemurafenib resistance partially. Nrf2-dependent glutathione metabolism plays a crucial role in the emergence of resistant cells.	MAPK-resistant melanomas are OXPHOS dependent, expressing Nrf2 downstream antioxidant response.	[31]
Li et al., 2018	C57BL/6 J male mice and melanoma B16-F10 cells	Mouse lung tissue with melanoma metastasis revealed a high expression of Nrf2 and its downstream antioxidant enzymes and inflammatory factors, including NF-κB P65, IL-6, and TNF-α.	The antioxidant response following oxidative stress plays a significant role in melanoma lung metastasis.	[32]
Sample et al., 2018	Human Skin Samples, A375 melanoma cell line, and WT/KO mouse embryonic fibroblasts	UVA induces P62 up-regulation in an Nrf2-dependent manner. Knockdown of Nrf2 prevents p62 up-regulation. Conversely, knockdown of P62 inhibits UVA-induced Nrf2 up-regulation. P62 overexpresses in melanoma and malignant melanoma independent of BRAF/NRAS mutation.	Nrf2 and P62 interact with each other in a positive feedback loop. The P62 overexpression contributes to tumor growth and metastasis.	[33]
Zhu et al., 2018	Female C57BL/6 J mice, Cell lines: B16-BL6, HPK, and HPM	Nrf2 and PD-L1 are overexpressed in melanomas and metastatic melanomas. UVA-induced PD-L1 upregulation is positively associated with Nrf2 activation.	UVR induces PD-L1 in an Nrf2-dependent manner, facilitating melanoma progression.	[34]
Arakawa et al., 2018	Human melanoma cell lines: CRL-1585 (C32), GAK, G-361, HMV-II, HMY-1, MeWo, MM-AN, SK-MEL-2, SK-MEL-31, and SK-MEL-28 murine melanoma B16BL6 cell line, and human non-small cell lung cancer H460 cell line	β-lapachone cytotoxicity in melanoma cell lines is dependent on the Nrf2/NQO1 axis activity. Carnosic acid induces NQO1 through further stabilization of Nrf2. Co-treatment of β-lapachone and carnosic acid increases the melanoma cell sensitivity to β-lapachone by elevating the expression of NQO1.	The NRF2/NQO1 axis can play a decisive role in improving the clinical response rate of NQO1-dependent anticancer therapies in malignant melanomas.	[35]
Aksenenko et al., 2019	Melanoma B16-bearing mice	The pro-oncogenic miR-155 is significantly elevated in the target organ of melanoma metastasis, downregulating NFE2L2 gene expression.	A reduction in NFE2L2 gene expression in the premetastatic organs may be associated with metastatic niche formation and stromal progression, results in premetastatic tissue reconstruction.	[36]
Chhabra et al., 2019	Neonatal human skin melanocyte	UVA and/or UVB exposure increase ROS and downregulate Nrf2 expression in melanocytes.	Nrf2-Keap1 pathway plays a key role in protecting skin cells against UVA and UVB induced oxidative damage.	[37]
Gagliardi et al., 2019	Human melanoma cell lines: A2058, A375, C8161, CHL-1, SK-Mel 5, SK-Mel 24, and MeWo	Activation of Nrf2 leads to a constant decrease in erastin-mediated AKR1C1÷3 expression. Moreover, inhibition of Nrf2 causes complete abolishment of ERA-stimulated CHAC1 expression. Nrf2 expression in tumor cells is lower than in normal tissue samples, without any important fluctuation during the stages of tumor progression. However, there is no association between Nrf2 expression and patient overall survival.	Melanoma cancer cells resistant to ferroptosis cell death efficiently activate Nrf2 and its downstream enzymes HO1.	[38]
Hämälaïnen et al., 2019	Human tissue samples, Cell lines: human primary melanoma IPC-298 (ACC 251), adult primary epidermal melanocytes (PSC-200-013), and metastatic melanoma SK-MEL-30	The Nrf2 mRNA decreases from benign lesions to primary melanomas. Moreover, the Nrf2 mRNA level varies in different stages of melanoma. High nuclear expression of Nrf2 protein can play a prognostic role in melanoma patients prior to nodal or distant metastasis. MiR-144-3p, miR-212-3p, miR-23b-3p, miR-340, and miR-93-5p appear to be related with Nrf2.	The miRNAs are associated positively with Nrf2 mRNA but negatively with its protein.	[39]
Kuo et al., 2019	Mouse epidermal JB6 P+ cell line, and human hepatocellular HepG2-C8	Nrf2 knockout mice developed multiple skin tumors following exposure to the carcinogens. Delphinidin prevents JB6 P+ cell transformation by epigenetic activation of the Nrf2-ARE pathway.	Redox imbalance predisposes cells to melanoma formation. Phytochemicals can play a chemopreventive role by inhibiting the neoplastic transformation of skin cells.	[40]
Estrela et al., 2019	Nude (nu/nu) male mice, Human A2058, COLO-679 and SK-Mel-28 melanoma cell lines	Pharmacologic silencing of glucocorticoid receptor (GR) reduces the expression of Nrf2 and antioxidant responses in metastatic B16-F10 melanoma cells, causing a drastic reduction of cell survival and tumor growth. The induction of Nrf2 overexpression reversed the anticancer effect of GR inhibition.	The cross-talk among GR, p53, and Nrf2 may be critical in BRAFV600E-mutated melanoma cells survival and metastasis. Combination of GR antagonists (mifepristone, RU486) with BRAF-related therapy could be a novel approach for improving the outcome, and preventing resistance to treatment in BRAFV600E-mutated metastatic melanoma.	[41]
Chipurupalli et al., 2020	C32, and SK-MEL-28 melanoma cell lines	STING activation downregulates the Nrf2 downstream antioxidant enzymes. diABZI induced STING-activation reduces proliferation, accelerates cell death, and prevents migration of melanoma cells by restricting the Nrf2 signaling pathway.	Increased activity of the Nrf2 in melanoma cancer cells may be due to a defect in STING activation.	[42]
Jasmer et al., 2020	Normal human embryonic melanocytes (NHEM), SK-Mel-2, SK-Mel-5, SK-Mel-28, and HEK293FT melanoma cell lines	Inhibiting Nrf2 or its downstream enzyme, HMOX1 reduces melanosphere formation in BRAF-driven melanoma cell lines.	Melanosphere formation has an HMOX-dependent mechanism correlating with tumorigenic capacity. HMOX1 contributes to drug resistance, melanoma invasion, and migration.	[43]
Jessen et al., 2020	A375, A-549, SK-MEL-2, SK-MEL-3, SK-MEL-28, UACC-62, UACC-257, M14, and LOXIMVI cell lines	Various internal and external signals activate Nrf2 in melanoma cells. siRNAs-dependent down-regulation of Nrf2 decreases the proliferation rate, promoting cell differentiation. Moreover, Nrf2 knockout prevents melanoma occurrence in vivo.	Activation of Nrf2 by intrinsic and extrinsic triggers present in the melanoma tumor niche results in developing a dedifferentiated phenotype and overproduction of COX2 and PGE2. This phenomenon creates an immune-cold tumor environment. Melanoma benefits from activating of the Nrf2 pathway.	[44]
De Cicco et al., 2021	The A375 human melanoma cell line	Cynaropicrin has an antioxidant effect; treated melanoma cells are less aggressive with lower clonogenic ability.	Cynaropicrin plays a chemopreventive role by inducing Nrf2 activation in melanoma cells.	[45]
Kreß et al., 2021	A375, LOXIMVI, M14, UACC-257, UACC-62, SK-MEL-2, and SK-MEL-28 cell lines	AKT activity was strongly reduced in NRF2-knockout cells in response to EGF induction compared to the control group, indicating the essential role of Nrf2 for full activation of EGFR. Nrf2 facilitates the expression of EGFR and its ligands, attributed to features such as invasion, tumor metastasis, and BRAFi resistance.	There is a positive feedback loop between Nrf2 and EGFR signaling pathway. The activation of that results in the survival and invasiveness of melanoma cancer cells.	[46]
Li et al., 2021	Skin melanoma tissue sample, the A375 human melanoma cell line	The amount of mGPDH protein in melanoma cells is significantly lower compared to normal skin cells, and metastatic melanoma compared to primary melanoma. Furthermore, the loss of mGPDH triggers Nrf2 expression without changing the NRF2 protein degradation, promoting melanoma migration and invasion.	mGPDH deficient cells are able to activate the Nrf2 signaling pathway, leading to melanoma progression and metastasis.	[47]
Liao et al., 2021	Normal skin cell line PIG1, melanoma cell lines: A375, G-361, HS1-CLS, MEL-CLS1-4, IGR-1, MEWO, NIS-G, WS1-CLS, and MML1	miR-130b-3p activates the Nrf2/HO-1 signaling pathway by inhibiting DKK1 expression in melanoma, leading to reduced melanoma cell ferroptosis.	The expression of miR-130b-3p reinforces activation of the Nrf2/HO-1 pathway followed by DKK1 suppression. The overexpression of Nrf2 correlates with the resistance of melanoma cells to ferroptosis.	[48]
Schmidlin et al., 2021	The HEK293, and A375 cell lines	FAM129B up-regulates Nrf2 by binding to Keap1 protein in A375 melanoma cells. Knockdown of FAM129B plays a protective role against melanoma metastasis.	Constitutive activation of Nrf2 induced by FAM129B potentiates BRAF mutant melanomas for metastasis.	[49]
Weitzenböck et al., 2022	Metastatic MCM1DLN, and Non-metastatic MCM1G lines from a patient with melanoma, metastatic WM1205Lu and Non-metastatic WM793b cell lines from ATCC.	Suppression of Nrf2 leads to a loss of ROS protection and promotes the epithelial-mesenchymal transition (EMT) phenotype, which is hallmarked by the expression of CD44. Cells devoid of NRF2 exhibit higher survival rates after a BRAF or an MYC inhibitor treatment.	Nrf2 silencing increases melanoma cell viability, invasiveness, and the risk of metastasis.	[50]
Wang et al., 2022	The A375, and A2058 cell lines	Following treatment with erastin and RSL3, the level of CAMKK2 mRNA was significantly elevated. Phosphorylation of CAMKK2 reduces during ferroptosis. Moreover, the phosphorylation of AMPK (known as a canonical substrate of CAMKK2) increases during ferroptosis. CAMKK2 prevents ferroptosis by repressing the production of lipid peroxidation in an Nrf2-dependent manner.	CAMKK2 regulates the AMPK–Nrf2 signaling pathway, leading to lower ferroptosis sensitivity of melanoma cancer cells.	[51]
Sanches et al., 2022	B16-F10 murine melanoma cell line	DTIC resistant cells showed a decreased level of Nrf2. However, the addition of metformin in DTIC resistance leads to increased Nrf2 and less resistance to DTIC. Moreover, the exclusive administration of metformin also reduces Nrf2 compared to control group, inducing cellular resistance to DTIC.	Metformin plays a dual role, inhibiting or promoting a DTIC-resistant phenotype in B16-F10 melanoma cells.	[52]
Feng et al., 2022	SK-MEL-28 human melanoma cell line	GSK3 expression in melanoma cells is enhanced by nobiletin, which is usually low in cutaneous melanoma. GSK3β inhibits the Keap1/Nrf2/HO-1 Signaling Pathway. The buildup of iron and ROS and the GSH depletion causes lipid peroxidation, thereby inducing ferroptosis.	Nobiletin stimulates ferroptosis in melanoma cells by GSK3β-mediated suppression of the Nrf2/HO-1 axis.	[53]
Li et al., 2022	A375 human melanoma cell line	Dimethyl fumarate (DMF) suppresses the Nrf2 antioxidant pathway as well as AKT/mTOR/ERK signaling pathways, causing ROS accumulation. The combination of DMF with vemurafenib significantly reduced melanoma cell growth and increased tumor cell death in vitro and in vivo.	A combination of DMF and vemurafenib could be a novel therapeutic method against melanoma	[54]
Argenziano et al., 2022	M14 melanoma cell line	The Nrf2 protein level was downregulated after the treatment of M14 cells with siNrf2-NB. Subsequently, the treated cells showed significant downregulation of the viability cells.	Nrf2 contributes to maintaining the chemoresistance in melanomas. siRNA-mediated inhibition of Nrf2 could be a promising strategy to overcome chemoresistance.	[55]

## Results

### Article selection

**Fig. 1:**
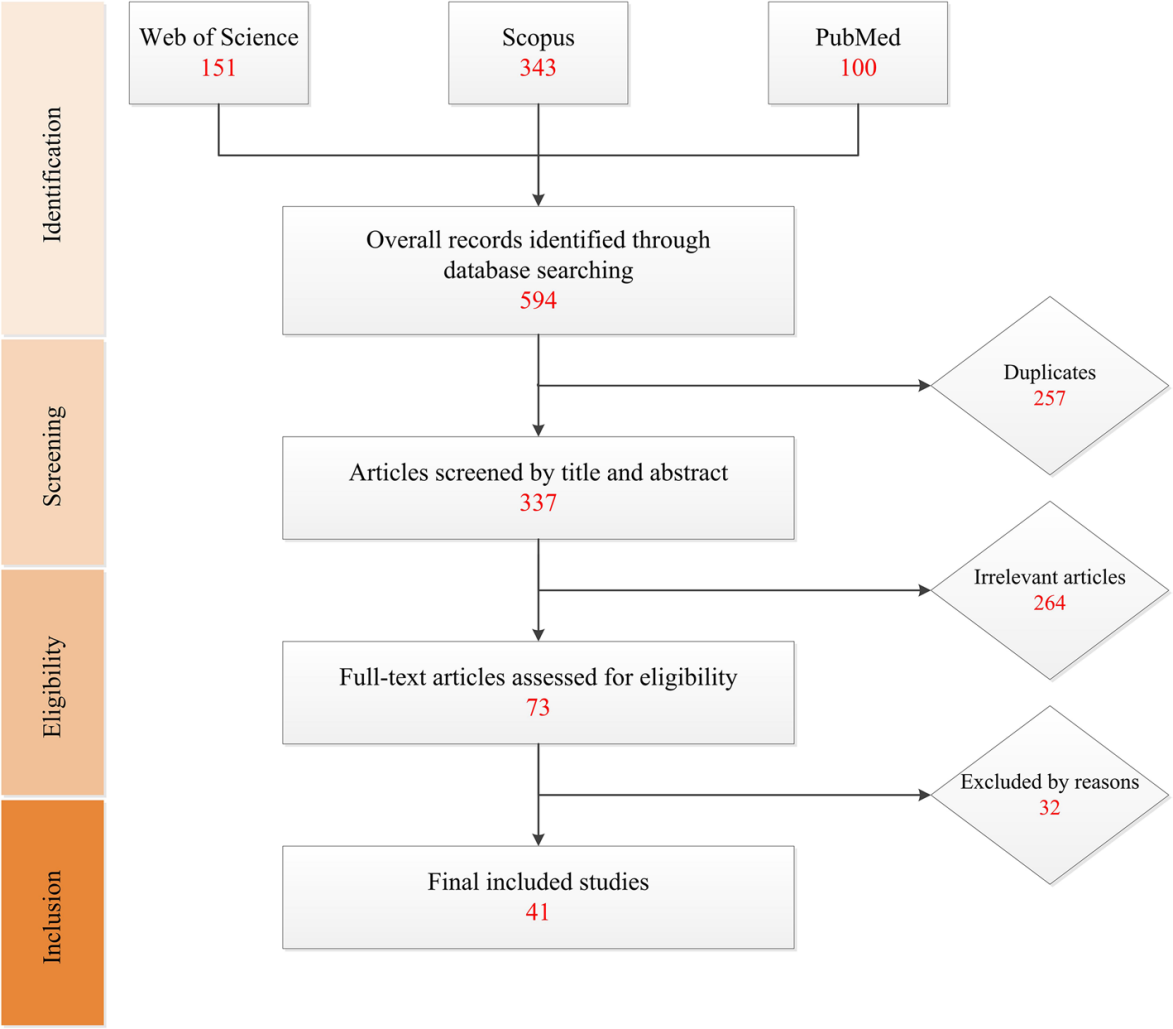
This flow diagram presents the process of study selection included in the qualitative systematic review

According to the search strategy we applied, the literature search provided 594 records, including 151 articles from WOS, 100 from PubMed, and 343 from Scopus. Removing duplicate reports resulted in 337 articles available for the initial assessment. We assessed the titles and abstracts and removed irrelevant articles, limiting our data to 73 articles for full-text review. Out of 73, 32 were excluded based on inaccessibility and exclusion criteria. Eventually, data were collected from 41 articles presenting the role of Nrf2 in melanoma initiation, progression, and metastasis (Fig. 1).

### Tabulation

All data extracted from the final articles are summarized in Table 1. All of the articles chosen for this study were published after 2014. Studies are mainly performed on human cancer cell lines, whereas others have focused on murine melanoma cell lines, mice, or human tissue samples.

#### Expression and activation of Nrf2 in melanoma

Due to UV radiation and melanin production, melanocytes create more ROS than surrounding keratinocytes in the skin. Evidence shows that oxidative stress, increased ROS level, and redox imbalance are critical parameters in melanogenesis. Indeed, the entire process of melanoma formation relies on oxidative stress and antioxidant response. Because of their more significant metabolic needs, melanoma cells exhibit even higher ROS and antioxidant responses than normal melanocytes [21, 56]. Melanocytes use adaptive strategies to withstand oxidative stress by expressing antioxidant scavenging activity. As a result, it has been proposed that targeting melanoma cells’ addiction to greater redox capability could be a suitable and effective treatment method. The master stress response transcription factor Nrf2, which regulates the expression of antioxidant enzymes such as HO-1, SOD, CAT, and GPX, is primarily responsible for redox homeostasis in melanoma [57]. Investigations on the expression of Nrf2 and its target genes have produced conflicting results. Even some studies reported no significant differences in Nrf2 expression between normal and melanoma cells [16].

Taken together, it seems that lower Nrf2 expression favor melanogenesis, and is appeared in early stages of melanoma. Nrf2 mRNA and protein levels are diminished during the early phases of melanoma formation. Even though Nrf2 is expressed in normal skin cells, it is considerably reduced in skin malignancies such as malignant melanoma, indicating that Nrf2 expression is linked to the incidence of skin cancers. However, higher Nrf2 expression in cancerous cells is linked to more aggressive and resistant melanomas, and poor prognosis [15, 39]. It is hypothesized that Nrf2 contributes in melanoma phenotypic switch between a proliferative and an invasive phenotype. The expression of genes linked to the melanoma progression change subsequent of Nrf2 expression. For example, Nrf2 inhibits the MITF (microphthalmia-associated transcription factor), the primary transcriptional regulator of the phenotypic transition in melanoma, and its expression changes through the melanoma progression. MITF down regulation causes invasive dedifferentiated melanoma, while overexpression causes a proliferative phenotype. It is plausible that Nrf2 expression is required during periods of fast development but is repressed once an invasive phenotype has emerged [11].

When melanoma advances, Nrf2 expression rises. Melanoma redox capacity is assumed to represent a spectrum ranging from low in normal cells to moderate in drug-sensitive melanomas and to high in drug-resistant melanomas [57]. Consequently, the expression of Nrf2 changes in benign lesions and progresses to primary and metastatic melanomas [39]. Although Nrf2 expression is higher in metastatic lesions than in primary sites, cytoplasmic Keap1 expression is lower, indicating that Nrf2 accumulates in the nucleus, leading to up-regulation of Nrf2 target genes [28]. For example, HO-1 expression has been found to be increased in B16-F10 murine melanoma cells as well as in-vivo melanoma tumor models, which has been linked to the higher proliferation rate [57].

BRAF (B-rapidly accelerated fibrosarcoma) and NRAS (N-Rat sarcoma virus) mutations, which are found in 50 and 20% of cases, respectively, are commonly responsible for dysregulation of the MAPK (mitogen-activated protein kinase)-proliferation signaling pathway in melanomas [58]. A direct link between Nrf2 activation and MAPK signaling has recently been discovered. The activation of the MAPK pathway causes Nrf2 to be expressed. BRAF/NRAS mutant melanoma cells activate the Nrf2 signaling pathway to protect cells against oxidative stress [20, 49]. BRAF inhibitors (BRAFi), like vemurafenib, suppress Nrf2 expression in BRAF mutant melanoma cells, although the effect is not significant, suggesting that other oncogenes also trigger the Nrf2 signaling pathway [20]. This is worth noting that BRAFi-resistant cells have higher Nrf2 expression than non-resistant cells [57]. However, not all Nrf2-dependent genes react the same way to BRAFV600E expression [43].

UV irradiation is also a determining factor in the expression of Nrf2 and subsequent antioxidant target genes. UV exposure enhances the Nrf2 expression and nuclear accumulation in a dose-dependent manner [17, 30, 59]. However, the effect of UV radiation on Nrf2 expression is still a matter of contention; some studies have reported no effect or even a negative effect [29]. GSH (glutathione) depletion and ROS accumulation caused by UVA (ultraviolet A) irradiation may trigger melanogenesis in people with defective Nrf2. Although Nrf2 silencing did not affect melanogenesis in the absence of UVA exposure, Nrf2-knockout cells exhibit higher tyrosinase activity and melanin content [21].

#### Nrf2 in melanoma progression

Nrf2, a crucial activator of antioxidant and phase two defenses in cells, has a dual role in melanoma. Although regular Nrf2 expression has a cytoprotective role against carcinogenesis, Nrf2 overexpression boosts antioxidant responses and changes the cellular redox state. In this aspect, antioxidants have been demonstrated to aid melanoma cell survival, proliferation, and tumor growth [44]. Nrf2 activity in transformed cells lowers ROS and inhibits tumor growth in vivo by mechanisms such as apoptosis sensitization and a reduced angiogenic/hypoxic response via HIF-1(hypoxia-inducible factor-1) instability and VEGFA (vascular endothelial growth factor A) suppression [16]. Nrf2 activators like cynaropicrin can reduce the clonogenic ability of metastatic melanomas in a dose- and time-dependent manner while not affecting normal melanocytes. Anti-apoptotic proteins such as XIAP (X-linked inhibitor of apoptosis protein) and Bcl-2 (B cell lymphoma-2) are also reduced in response to cynaropicrin treatment [45]. Another study discovered that delphinidin, an Nrf2 epigenetic enhancer, suppresses JB6P^+^ cell transformation, implying that delphinidin could be employed as a chemopreventive medication [40]. However, Nrf2 activators or inhibitors should be utilized carefully and in line with the stage of the disease in order to be effective.

UV-induced aggregation of Nrf2 in B16-F10 cells was found to diminish cell viability and increase caspase-3 expression, a key indicator of apoptosis. Surprisingly, ionizing radiation in Nrf2 silenced cells synergistically affected melanoma cell viability, elevating apoptosis [30]. This dual behavior may be due to the complex web of interactions around Nrf2. As Arslanbaeva et al. indicated, in vitro and in vivo, the combination of brusatol, a potent Nrf2 inhibitor from the plant Brucea javanica, and UVA irradiation could reduce the melanoma cell proliferation and induce apoptosis in melanoma cells [57].

In melanoma, the activity of receptor tyrosine kinases (RTKs) is normally restricted. Some RTK family members like EGFR may be expressed in MITF-low melanoma cells, and under this circumstance, the EGFR expression is linked to a pro-invasive and pro-metastatic melanoma phenotype with resistance to BRAF/MEK inhibitors. Stress-induced Nrf2 mediates the expression and activity of EGFR by increasing the EGFR levels and its ligands like EGF and TGFα. A canonical ARE is found in the EGF promoter, and Nrf2 binds to it directly, leading to a higher level of EGF for AKT activation. Furthermore, Nrf2 blocks the MITF activity, the melanocytic lineage factor responsible for suppression of EGFR and TGFA. Hence, Nrf2 indirectly derepresses the EGFR, contributing in the maintenance of EGFR-expressing melanomas [46]. Reversely, EGFR stimulates Nrf2, providing a positive feedback loop. In vivo Nrf2 silencing has been shown the low EGFR expression and proliferation rate, preventing melanoma growth, and increasing tumor-free survival. However, Nrf2-induced suppression of MITF activity also contributes to development of dedifferentiated, invasive phenotype in another ways. The antigenicity of melanoma cells is significantly influenced by the proteins expressed by MITF target genes. Nrf2-mediated down regulation of pigmentation markers (melanoma antigens) due to MITF inhibition promote a dedifferentiated melanoma cell that can escape from immune recognition [44]. Another study has demonstrated that inhibiting Nrf2 by pharmacologic activation of the Nrf2’s upstream gene reduces proliferation, accelerates cell death, and slows melanoma progression [42]. Inhibiting either Nrf2 or its target gene, HMOX-1 (heme oxygenase 1 gene) prevents melanosphere formation, an indicator of cell tumorigenic potential, in melanoma cell lines. Moreover, HMOX-1 overexpression in A375 melanoma cells increases tumor volume, whereas silencing HMOX-1 reduces tumor growth in mouse models [43].

The cellular redox status has been shown to alter the innate immune responses. Albeit this has yet to be determined, Nrf2 may cooperatively modulate the innate immune response, repressing the pro-inflammatory mediators’ expression by mediating the transactivation of antioxidant and other cytoprotective genes [60]. Nrf2 depletion can increase the susceptibility to infection and inflammatory diseases due to impaired cellular stress response and exacerbated immune-mediated hypersensitivity and autoimmunity [61]. Nrf2 can also aid anti-tumor immunity by inducing immune-dependent cascades such as IL-17D-dependent NK (natural killer) cell recruitment early in carcinogenesis before the pro-tumor action of Nrf2 manifests. However, induction of IL-17D at advanced stages is not always associated with a good prognosis [23].

There is evidence that Nrf2 has a significantly greater tendency to suppress immunity, mostly in advanced stages. BRAF wild-type melanomas have been treated with monoclonal antibodies that target immune checkpoint proteins such as anti-PD-1 (programmed cell death protein-1) (like pembrolizumab or nivolumab). Nrf2 inhibits the recruitment of interferon-secreting immune cells, reducing PD-L1 (programmed death-ligand 1) expression in the process. Given that increased PD-L1 expression has been linked to enhanced anti-PD-1 therapeutic effectiveness, this might explain why Nrf2 overexpression is linked to immunotherapy resistance [11]. Nrf2 has also been found to have a direct impact on PD-L1 expression. Zhu et al. found that Nrf2 stimulates PD-L1 transcription, and targeted Nrf2 inhibition is an alternative way of suppressing PD-1/PD-L1 to trigger tumor infiltration by CD4^+^ and CD8^+^ T lymphocytes and consequentially restrict melanoma growth [34].

Cytosolic DNA induced from nuclear/mitochondrial DNA damage in cancer triggers the activation of cGAS-STING (cyclic GMP-AMP synthase-stimulator of interferon genes) pathway. The recruitment of cGAS-STING pathway plays an important role in tumor immunogenicity, and Its activation has been demonstrated to reduce mice’s resistance to PD-1 blockage [62, 63]. Evidence exists to show the suppressive role of Nfr2 on cGAS-STING pathway, providing an immune-cold microenvironment [44, 64]. Moreover, Nrf2 is demonstrated to be a potent inducer of COX2, the enzyme that facilitate the production of PGE2. Following this, the PGE2 contributes to the suppression of the innate immune response and the attenuation of T cell receptors, establishing an immune-evasive tumor microenvironment [64, 65]. Nrf2 inhibitors are thus expected to function in conjunction with checkpoint inhibitors in the treatment of melanoma. Such a combination is presently not possible due to the lack of potent Nrf2 inhibitors. Nevertheless, preclinical studies have shown that COX inhibitors and STING agonists work in conjunction with anti-PD-1 immunological therapy, indicating that targeting Nrf2 downstream indicators may be beneficial [42, 44].

**Fig. 2:**
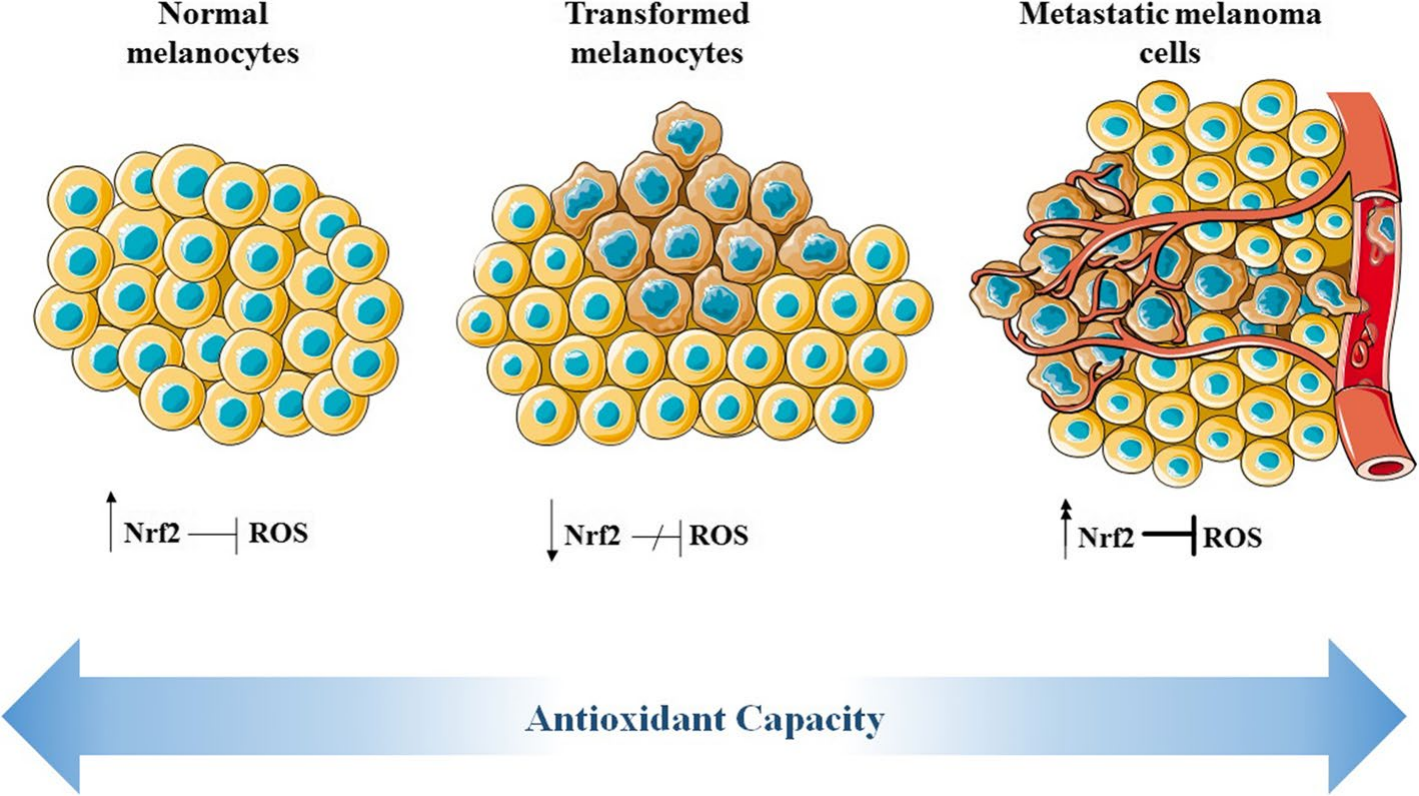
Nrf2 plays a dual function in melanoma, depending on the cell stage. The standard expression of Nrf2 has a protective role against oxidative stress and cell damage, maintaining normal melanocytes. Nrf2 depletion and ROS accumulation make cells susceptible to transformation and cancer initiation. Evidence exists to show that reduced Nrf2 expression is associated with the incidence of melanoma. On the contrary, aberrant overexpression or constitutive activation of Nrf2 leads to invasive, metastatic, and radio/chemoresistant melanomas with poor prognosis. Indeed, after cell transformation has occurred, Nrf2 can become a pathway for cancer progression

Overall, it can be concluded that the onco-promoter/onco-suppressor function of Nrf2 varies by cell stage and is context-dependent. Furthermore, whereas transient Nrf2 activity protects against cancer, constitutive Nrf2 activation caused by Keap1 or Nrf2 genetic variations may promote cancer progression and tumor growth (Fig. 2).

#### Nrf2, and melanoma metastasis

There is evidence that Nrf2 activation enhances cancer cell metastasis in melanoma, suggesting that Nrf2 has an onco-promoter role [34, 47, 59]. In fact, as long as the cell is intact, Nrf2 and its lower genes try to save the cell from becoming cancerous by reducing oxidative stress. However, if the situation progresses in such a way that cell transformation occurs, the cancer cell uses Nrf2 in favor of itself, and higher expression of Nrf2 makes melanoma cells resistant to high levels of ROS and oxidative stress produced from cell metabolism. In this regard, Nrf2 is associated with invasiveness and pro-metastatic features. Studies have shown that the ROS or ROS-enhancing chemicals at this phase might be effective in cancer treatment, whereas antioxidants provide a more desirable environment for melanoma progression and metastasis [32, 45, 57, 66].

HMOX1 is one of the driving forces in melanoma invasion and migration. Silencing Nrf2, which causes HO-1 to be down-regulated, prevents melanoma cells from invasion and migration [43]. UV irradiation also has a significant role in regulating the apoptosis, migration, and invasion of melanoma cells in the Nrf2-dependent manner. Gao et al. found that downregulation of Nrf2 reduces the ability of radiation-exposed B16-F10 cells to invade and migrate. Surprisingly, however, overexpression of Nrf2 exhibited similar effects on cell survival, invasion, and migration, demonstrating Nrf2’s dual function in melanoma [30]. It has been discovered that inhibiting the function of Nrf2 by pharmacologically activating the STING pathway accelerates cell death and decreases melanoma cell migration, and has a synergistic effect with BRARi in cell death [42].

It has been shown that FAM129B, an Nrf2 inducer, also makes melanoma cells more susceptible to metastasis. Intriguingly, knocking down FAM129B or reducing its phosphorylation reduces melanoma cell invasion and migration in an Nrf2-dependent way [49]. In metastatic melanomas, mGPDH (mitochondrial glycerol-3-phosphate dehydrogenase) activity is reduced. Li et al. showed that in A375 melanoma cells, knocking down mGPDH activates the Nrf2 pathway, causing melanoma cell metastasis, whereas co-inhibiting Nrf2 prevents melanoma cell metastasis in vivo and in vitro. These findings show that via inhibiting the Nrf2 signaling pathway, mGDPH suppresses melanoma cell motility and invasion [47]. P62, an autophagy-degrading factor, is overexpressed in malignant and metastatic melanomas regardless of BRAF/NRAS mutant status. Overexpression of P62 has been shown to promote tumor growth and metastasis in various malignancies, including melanoma. P62 accumulates in melanocytes and melanoma cells due to different stimuli, including UVA [33, 49]. P62 interacts with Keap1, preventing it from inhibiting Nrf2 and boosting Nrf2. Surprisingly, p62 is a direct target of Nrf2, and the two are linked in a positive feedback loop. UVA-induced p62 upregulation is inhibited when Nrf2 is silenced. P62 knockdown, on the other hand, hinders Nrf2 overexpression. As a result, p62’s oncogenic activity may be influenced by Nrf2 antioxidant responses [49, 67].

Nrf2 has also been linked to establishing the metastatic niche and stromal progression in various studies. In the pre-metastatic liver of melanoma-bearing mice, a reduction in Nrf2 expression was discovered, leading to oxidative stress and acidosis. This state is consistent with the tumor microenvironment, enhances melanoma cell survival, and facilitates metastasis [36]. These findings imply that redox imbalance changes the environment in favor of metastasis and that the Nrf2 antioxidant response is critical in this process.

There is also evidence of the preventive role of Nrf2 in metastasis. The phytochemical cynaropicrin enhances the Nrf2 activity in melanoma cells. Cicco et al. proved that cynaropicrin reduces the capacity of human melanoma cells to migrate, invade, and proliferate in vitro. However, this phytochemical can also suppress MAPK/ERK (extracellular signal-regulated kinase) and NFκB (nuclear factor kappa-light-chain-enhancer of activated B cells) pathways in human melanoma cells. Thus we cannot consider the anticancer effects of cynaropicrin to be attributed only to Nrf2 stimulation [45]. Zhu et al. reported that B16-F10 melanoma cells lacking Nrf2 are more prone to tumor development and lung metastasis [24]. Weitzenböck et al. found that Nrf2 suppression leads to the expression of CD44 and the development of epithelial-mesenchymal transition phenotype. Of note, treatment of Nrf2 depleted cells with BRAF or MYC inhibitor results in a higher survival rate of melanoma cells [50]. This shows that antioxidant intervention could be a game-changing strategy for improving melanoma patient survival.

Taken together, it can be concluded that despite the contradictory findings regarding the role of Nrf2 in metastasis, most studies indicate the facilitative role of Nrf2 in this subject. Further investigation is necessary to find a clear conclusion as to whether or not nrf2 activity is metastatic.

#### Clinical role of Nrf2 in melanoma

Melanoma is a poorly responsive malignancy with a 5-year survival of about 15% based on conventional chemotherapy [68]. In recent years, novel targeted treatments and immunotherapy have drastically altered the general approach to melanoma treatment. BRAF and NRAS mutations in the MAPK signaling pathway are found in about half of all melanoma cases [58], resulting in constitutive activation of the MAPK pathway.

In patients with advanced mutant BRAF melanoma diseases, BRAFi like as vemurafenib or dabrafenib have been used successfully in conjunction with MEK (mitogen-activated protein kinase) inhibitors such as trametinib. Moreover, for BRAF wild-type melanomas, current recommendations advocate using monoclonal antibodies (i.e., ipilimumab) that target immunological checkpoint proteins such as anti-PD-1 or CTLA-4 (cytotoxic T-lymphocyte antigen 4) in combination with anti-PD-1 therapy [69].

Despite the fact that new agents for melanoma have improved survival compared with conventional chemotherapy, the response rate of metastatic melanoma is still low and has little influence on prognosis. Malignant melanoma cells’ extensive resistance typically limits modern treatments’ efficacy. Adaptive processes of melanoma cells allow them to offset the effects of medicines, resulting in a multidrug-resistant phenotype [70]. Melanoma cells rewire their proliferation and survival pathways, putting their inherent resistance to apoptosis and ferroptosis to work, making them ‘bulletproof’ against a wide range of chemotherapeutic medicines [71]. Identifying signaling pathways involved in the regulation and execution of apoptosis and altering them to promote melanoma cell sensitivity has offered a unique method for battling melanoma chemoresistance in recent years. In this context, a mountain of evidence suggests that metabolic modification plays a significant role in melanoma cells’ chemo- and radio-resistance. During melanoma progression, metabolic reprogramming, including redox status, results in constitutive activation of Nrf2 and its downstream enzymes. As a result, intracellular ROS levels drop considerably, conferring chemoresistance [18, 72]. Therefore, Nrf2 is critical for controlling the redox system and fostering melanoma resistance cells through mediating downstream antioxidant enzymes, detoxifying and drug-metabolizing enzymes, drug transporters, and multidrug resistance proteins [73]. Although melanoma cells often create reactive oxygen species, the co-evolution of adaptive systems, specifically Nrf2 as a master regulator, makes them more sensitive to emerging chemoresistant cells’ ROS tolerance. In vitro studies have shown that suppressing Nrf2 can make melanoma cells more sensitive to anticancer medicines. As expected, Nrf2 devoid cells have increased ROS levels, making them more susceptible to anticancer drugs such as dacarbazine (DTIC) or cisplatin [18, 55]. Thus targeting Nrf2 may prevent melanoma from further progression and chemoresistance. Nevertheless, whether targeting Nrf2 alone is more beneficial or detrimental is still debated. If Nrf2 is targeted as a single therapy, melanoma phenotypic change will likely occur. Thus A combinatorial strategy would very certainly be required [11].

Drugs that reduce the corticosterone production or its effect on the receptor indirectly downregulate Nrf2, preventing tumor growth. In contrast, the induction of Nrf2 overexpression reversed this anti-tumor effect [26, 41]. According to the Rocha study, TMZ (temozolomide) promotes Nrf2 activation, which leads to the expression of the GCLM (glutamate-cysteine ligase modifier) and GST (glutathione S-transferase) genes. Melanoma cells eventually develop TMZ resistance due to high levels of GSH. GSH depletion, on the other hand, boosted TMZ sensitivity in cells [25]. As a result, using TMZ in conjunction with Nrf2/GSH inhibitors enhances therapy outcomes.

According to multiple studies, RAF/RAS isoforms activate the Nrf2 pathway, and BRAF/NRAS mutant melanomas express more Nrf2 [57, 74]. Melanoma cells also accumulate more Nrf2 in their nuclei, much higher in MAPKi-resistant cells. Moreover, MAPKi-resistant melanomas exhibit higher oxidative phosphorylation and oxygen consumption in their mitochondria. As a result of the robust activation of the Nrf2 signaling pathway, these cells can modify mitochondrial metabolism, allowing MAPKi-resistant cells to survive. In BRAFi-resistant cells, Nrf2 silencing improves the efficacy of treatments like vemurafenib or PLX4032 [31, 43, 54, 75]. These findings imply that resistant melanoma cells rely on the mitochondrial oxidative phosphorylation system (OXPHOS) and that Nrf2-dependent glutathione metabolism is essential for the development of refractory melanomas. Thus, the activation of the Nrf2 pathway is closely linked to the development of resistant melanomas [31].

However, the effect of Nrf2 inhibitors on tumor growth suppression cannot yet be conclusively theorized. Studies have revealed that adding metformin besides the main chemotherapeutic agents has a promising effect in preventing chemoresistance in an Nrf2-dependent manner. Sanches et al. investigated how metformin affects oxidative stress and how it affects DTIC resistance in B16-F10 murine melanoma cells. They found that when melanoma cells were first pre-treated with metformin before and during the induction of DTIC resistance, metformin synergized with DTIC and the cells became sensitive to the therapy. This effect does not occur in the exclusive treatment with metformin or DTIC, and both of these conditions lead to the chemoresistance. According to findings, sensitive cells pre-treated with metformin and DTIC had an increased level of Nrf2 compared to the low Nrf2 levels of resistant cells [52]. Moreover, the cytotoxicity of some therapeutics depends on the activation of the Nrf2/NQO1 (NAD(P)H Quinone Dehydrogenase 1) axis. Co-treatment of melanoma cell lines with an Nrf2 inducer and β-lapachone increase the cytotoxic effect of β-lapachone on the melanoma cells [35].

Ferroptosis is a kind of programmed cell death accompanied by lipid peroxidation and iron accumulation, vital for tumor growth control. It has been shown that activating the Nrf2 signaling pathway can prevent ferroptosis cell death, boosting tumor development and resistance to chemotherapy. Thus, melanomas with a high Nrf2/HO-1 activity are resistant to ferroptosis cell death. The Nrf2/HO-1 signaling pathway impresses melanoma cell chemosensitivity by modulating microRNAs and ferroptosis-related proteins. For instance, activation of AKR1C1-3 (Aldo-keto reductase family 1-member C1-3) inhibit ferroptosis by lowering lipid ROS levels in melanoma cells. Nrf2 enhances elastin-mediated AKR1C1-3 production, whereas Nrf2 inhibition prevents erastin-stimulated CHAC1 (cation transport regulator-like protein 1) expression during ferroptosis induction and inhibits ferroptosis-induced AKRs (Aldo-keto reductases) expression [30, 38, 66]. Liao et al. reported that the miR-130b-3p activates Nrf2/HO-1 pathway by targeting Dickkopf1, inhibiting ferroptosis cell death in melanoma cells [48]. Therefore, inducing ferroptosis following Nrf2 suppression has the potential to overcome tumor cell apoptosis resistance. Nobiletin, a natural product extracted from citrus peel, can induce ferroptosis by GSK3β-mediated downregulation of the Nrf2/HO-1 axis [53].

#### The prognostic role of Nrf2 in melanoma

Constant Nrf2 activation, as previously stated, promotes tumor development and metastasis, resulting in a weak prognosis [59]. Many studies have linked greater Nrf2 expression in melanoma cells to a more aggressive phenotype and a worse prognosis [20, 27, 57, 64]. A greater Clark level, a deeper Breslow index, and nodular development are linked to increased Nrf2 expression. Furthermore, higher Nrf2 expression can predict patient survival before nodal or distant metastases [39]. Hintsala et al. reported that nuclear NFE2L2 expression is also linked to melanoma cell distant metastasis, nodular histology, and a deeper invasion, indicating that Nrf2 has a predictive function in original melanomas [20, 28]. Patients with melanoma cells expressing nuclear Nrf2 have a worse survival rate [57]. Hence, genetic manipulation or pharmacologic inhibition of Nrf2 may enhance melanoma patient survival in advanced stages. These findings support the pharmacological use of Nrf2 inhibitors such as ML385 and brusatol to avoid distant melanoma spread and treat metastatic melanomas. Although these drugs have drawbacks, they are effective [26, 41, 47].

Unlike other studies, one study found that lower Nrf2 expression is associated with a worse outcome in cutaneous melanomas. Oncogene-induced decrease of Nrf2 is thought to be an adaptive mechanism for establishing a pro-inflammatory state that promotes cell survival and proliferation, according to researchers [16].

## Conclusion

Nrf2, the master regulator of the antioxidant defense system, is expressed in melanocytes and malignant melanoma cells. The Nrf2 expression level is associated with the incidence of melanoma and co-opted during cancer progression. Indeed, the dual function of Nrf2 is context-dependent and varies by cell stage. Nrf2’s activity as a cellular defender at an early stage of melanogenesis is likely to be transformed into a cancer driver by oncogenic alterations that promote cell survival and provide protection against oxidative stress. Nrf2 addicted cancer cells exhibit more aggressive disease and poor prognosis. Thus a potential treatment option might target the disturbed redox homeostasis present in melanoma. However, it is becoming clear that treating melanomas by reversing increased oxidative stress is a misreading of the tumors’ complex altered redox homeostasis. Targeting Nrf2 combined with conventional anti-melanoma treatments like MAPK inhibition or immunotherapy may be more beneficial.

Nevertheless, there are conflicting data on the role of Nrf2 in melanoma; Lack of sufficient knowledge makes an emerging goal in melanoma research to elucidate the underlying mechanism of Nrf2 activity and its function in this cancer.

## Data Availability

The datasets used in this investigation can be found in the paper.

## Abbreviations

ARE: Antioxidant response element
AKR: Aldo-keto reductase
AKR1C1-3: Aldo-keto reductase family 1-member C1-3
BRAF: B-rapidly accelerated fibrosarcoma
Bcl-2: B cell lymphoma-2
DTIC: Dacarbazine
CAT: Catalase
cGAS: Cyclic GMP-AMP synthase
CHAC1: Cation transport regulator-like protein 1
COX2: Cyclooxygenase 2
CTLA-4: Cytotoxic T-lymphocyte antigen 4
EGFR: Epithelial growth factor receptor
GCLM: Glutamate-cysteine ligase modifier
GPX: Glutathione peroxidase
GSH: Glutathione
GST: Glutathione S-transferase
HIF-1: Hypoxia-Inducible Factor-1
HMOX-1: Heme oxygenase 1 gene
HO-1: Heme-oxygenase-1
Keap1: Kelch-like ECH-associated protein
MAPK: Mitogen-activated protein kinase
MEK: Mitogen-activated protein kinase
MITF: Microphthalmia-associated transcription factor
NRAS: N-Rat sarcoma virus
NFE2L2: Nuclear Factor Erythroid 2 Like 2
Nrf2: Nuclear factor erythroid2-related factor
OXPHOS: Oxidative phosphorylation system
PD-1: Programmed cell death protein 1
PD-L1: Programmed death ligand 1
PGE2: Prostaglandin E2
ROS: Reactive oxygen species
RTK: Receptor tyrosine kinase
SOD: Superoxide dismutase
STING: Stimulator of interferon genes
TMZ: Temozolomide
UV: Ultraviolet
VEGFA: Vascular endothelial growth factor A
WOS: Web of science
XIAP: X-linked inhibitor of apoptosis protein
